# Evaluation of the Effect of Nanoparticle Graphene Oxide on Flexural Strength of Glass Ionomer Cements

**DOI:** 10.1155/2023/8183167

**Published:** 2023-01-30

**Authors:** Farahnaz Sharafeddin, Hajar Farhadpour, Reza Hefzollah

**Affiliations:** ^1^Department of Operative Dentistry and Biomaterials Research Center, School of Dentistry, Shiraz University of Medical Sciences, Shiraz, Iran; ^2^Department of Operative Dentistry, School of Dentistry, Shiraz University of Medical Sciences, Shiraz, Iran; ^3^School of Dentistry, Shiraz University of Medical Sciences, Shiraz, Iran

## Abstract

**Aim:**

Glass ionomer (GIC) is a widely used restorative material in dentistry, but it has relatively weak mechanical properties. In this research, the effect of graphene oxide (GO) on the flexural strength of GIC was investigated.

**Materials and Methods:**

In this experimental study, 60 GIC samples in 6 groups of 10 were prepared, including Group 1: control conventional glass ionomer (CGIC), Group 2: CGIC + 1% wt of GO, Group 3: CGIC + 2% wt of GO, Group 4: control resin-modified glass ionomer (RMGI), Group 5: RMGI + 1% wt of GO, and Group 6: RMGI + 2% wt of GO. The samples were kept for 24 hours. The flexural strength of the samples was measured by using a universal testing machine. Data were analyzed by two-way ANOVA and posthoc Tukey test. (*P* < 0.05).

**Results:**

In the RMGI groups, the mean flexural strength value of the RMGI + 2% GO group was significantly higher than that of the RMGI control group (*P*=0.027). In the comparison of RMGI groups with their corresponding CGIC groups, the mean flexural strength values of all RMGI groups were significantly more than CGIC groups (*P* < 0.001). RMGI + 1% GO was not significantly different from control RMGI and RMGI + 2% GO (*P*=0.802, *P*=0.395, respectively). There was no significant difference between CGIC groups.

**Conclusion:**

Adding 2% by weight of GO to RMGI increases the flexural strength of RMGI, which could be of great importance in clinical practice in order to reinforce the mechanical properties of this dental material. The flexural strength of RMGI is higher than that of CGIC.

## 1. Introduction

Glass ionomer cement (GIC) is a material with a self-adhesion feature. GICs are classified as materials known as acid-based cement. They are based on the product of reacting weak polymer acids with powdered glass. Chemically, this consists of a combination of fluoroaluminosilicate glass powder and liquid polyacrylic acid [1]. GIC was first described by Wilson and Kent in 1972 and has since developed progressively to enhance its properties and expand its uses. It is used for cementing fixed dental prostheses, as liners or bases to restore carious and noncarious lesions, as core build-up material, orthodontic bands and brackets, as pit and fissure sealant, and for atraumatic restorative technique (ART) [2].

Graphene oxide (GO), a two-dimensional new carbon material, is regarded as an attractive biomaterial due to its unique lamellar structure, large specific surface area, and a large number of oxygen-containing functional groups (hydroxyl, carboxyl, etc.) [3].

Based on the research conducted on GO in the field of dentistry, this material has had a significant effect on improving implants, improving bleaching materials, helping tissue engineering, preventing the formation of bacterial biofilm, and improving the properties of resins [4]. GO has exhibited promising properties including mechanical strength, chemical stability, and thermal stability, along with antibacterial properties through combination with adhesives. GO functional groups modify the surface's chemical properties through complex surface reactions. In addition, it has been reported in a study that GO acts to reduce the risk of secondary caries and increase bond strength [5]. GO has good antibacterial effects due to its lamellar structure and sharp edges on a molecular scale, which can cause physical damage to cell membranes and cause oxidative stress reactions, which can possibly weaken bacterial resistance [6].

One of the most prominent features of GIC is its fluoride release [7]. There are various advantages of GICs, such as chemical bonding to the tooth structure, thermal compatibility with the tooth structure, tooth-colored restorative material, mild pulp response, and caries preventive action by releasing fluoride [8–10]. These features make GIC a desirable dental restorative material. But some disadvantages such as sensitivity to moisture during its setting, undesirable esthetics due to a lack of translucency, and especially poor mechanical properties, compromise its advantages [7, 11]. Therefore, many modifications have been conducted in order to eliminate these disadvantages [1].

Flexural strength is considered one of the most important factors of mechanical properties. As no studies were conducted to evaluate the effect of GO addition on the flexural strength of the different GICs contained, this study was carried out. The null hypothesis tested that GO nanoparticles have no effect on flexural strength of GICs.

## 2. Materials and Methods

The Research Ethics Committee of Shiraz University of Medical Sciences (IR.SUMS.DENTL.RES.1401.022) approved this study.

### 2.1. Preparation of GO/GIC Mixture

To determine the desired weight percentage, which is 1% and 2%, a scale with an accuracy of ±0.0001 grams (A&D, GR + 360, Tokyo, Japan) was used. By using this device at room temperature, the desired amount of nano-GO (KNV's Incorporation, Maharashtra, India) was prepared, and these amounts were mixed with GIC powder. After adding GO to GIC powder, first, the mixture was mixed manually and then the mixture was placed on a vibrator for optimal mixing.

### 2.2. Samples Preparation

60 samples of conventional GIC (CGIC) (GC Corporation, Japan) and resin-modified glass ionomer (RMGI) (GC Corporation, Japan) were prepared in the following groups (*n* = 10):

- Group 1: CGIC samples (control group)
- Group 2: samples of CGIC + 1% GO
- Group 3: samples of CGIC + 2% GO
- Group 4: RMGI samples (control group)
- Group 5: samples of RMGI + 1% GO
- Group 6: samples of RMGI + 2% GO

#### 2.2.1. Preparation of CGIC Samples

According to the manufacturer's instructions, a scoop of CGIC powder, containing 0, 1, and 2% GO, was mixed with a drop of liquid for 25 seconds. Mixing was done on the surface of a cold glass slab using a plastic spatula. The samples were then placed in the mold (with a gap in the central part (25 × 2 × 2 mm)) for 5.5 min while the top surface of the samples was covered with a transparent matrix celluloid strip (Fintrec, M-TP, PulpdentCorporation, Watertown, MA, USA) to completely cure the GIC.

#### 2.2.2. Preparation of RMGI Samples

**Figure 1:**
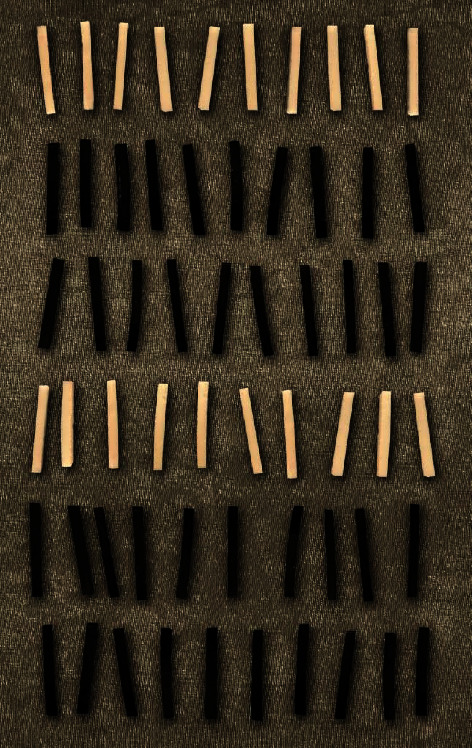
All prepared samples.

According to the manufacturer's instructions, a scoop of RMGI powder, containing 0, 1, and 2% graphene oxide (14, 15, 16, and 17), was mixed with two drops of liquid for 25 seconds. The mixture was transferred to the mold, and the upper surface of the samples was covered by a transparent matrix celluloid strip. Then, the upper surface of the samples was cured in three parts for 20 seconds using a light-cure unit (Monitex, Bluelux, GT 1200, Taiwan) attached to a glass plate with an intensity of 1200 mW/cm^2^. After removing the sample from the mold, its bottom surface was cured in the same way. All prepared samples are shown in Figure 1.

The brand names, manufacturer, and chemical composition of the materials are presented in Table 1.

The samples were placed in an incubator (Nuve, Turkey) for one day at a temperature of 37 degrees and relative humidity of 100% in order to complete the setting.

### 2.3. Flexural Strength Test

**Figure 2:**
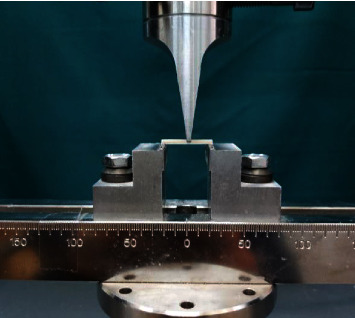
Universal testing machine.

After this period, the samples were tested to measure the flexural strength in a universal testing machine (ZWICK/ROELL ZO20, Germany) shown in Figure 2, with a crosshead speed of 0.5 mm/min. The flexural strength (*σ*) was calculated in megapascals (MPa) using the following equation:

(1) $$\begin{array}{c} \sigma =\frac{3FL}{2BH}\text{,}\\ E=\left(\frac{FL\;3}{4BH}\right)3d\text{,} \end{array}$$

where *F* is the maximum load (*N*), *L* is the length of the sample (mm), *B* is the width of the sample (mm), *H* is the height of the sample (mm), and *d* is the deflection (mm) corresponding to the load *F*.

### 2.4. Data Analysis

Data were analyzed using SPSS version 23 (SPSS Inc., IL, US). Two-way ANOVA analysis and posthoc Tukey test were used to show the effects of GI type and GO percentage on flexural strength.

## 3. Results and Discussion

### 3.1. Results

**Figure 3:**
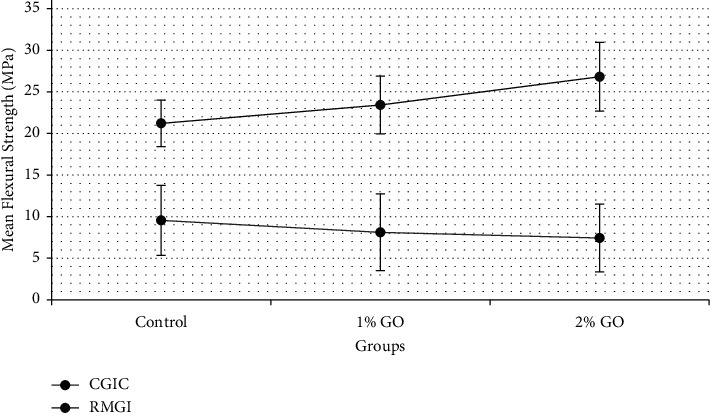
Mean flexural strength values. The error bars represent the standard deviation of measurements for 10 samples (*n* = 10).

Two-way ANOVA and posthoc Tukey tests showed that, in comparison to RMGI groups with their corresponding CGIC groups, the mean flexural strength values of all RMGI groups were significantly higher than those of CGIC groups (*P* < 0.001). Control CGIC was not significantly different from CGIC + 1% GO and CGIC + 2% GO (*P*=0.963, *P*=0.832, respectively). There was no significant difference between CGIC + 1% GO and CGIC + 2% GO (*P*=0.999). Between RMGI groups, the RMGI + 2% GO group's mean flexural strength value was significantly higher than the RMGI control group (*P*=0.027). However, RMGI + 1% GO was not significantly different from control RMGI and RMGI + 2% GO (*P*=0.802, *P*=0.395 respectively). The mean flexural strength values and standard deviation (SD) of each value are summarized in Table 2 and Figure 3. Moreover, posthoc Tukey test results are summarized in Table 3.

### 3.2. Discussion

Several studies have been conducted to improve the brittleness, physicomechanical properties, and moisture sensitivity of GICs through the addition of reinforcing fillers like polyethylene fiber, hydroxyapatite, and chitosan to the GIC powder [12–14]. The key goal of this investigation was to improve the physical properties of GICs using GO, a derivative of graphene.

As a measure of material strength, the flexural test was considered the most appropriate. In GIC, planes of atoms can only be separated (for example, in tensile failure) or they can slip (for example, in shear failure). Since flexural strength develops tensile, compressive, and shear stresses during the test, it was chosen as the main indicator of GIC physicomechanical properties in this study. By testing the material under its most challenging mechanical condition, we may be less inclined to accept a material that fails prematurely because of insufficient strength [15–17].

In previous studies, different amounts of GO (0.5, 1, 2, 3, and 4 wt%) were tried in different materials [15, 18–20]. The researchers found that adding 0.5, 1, or 2 percent GO improved the mechanical properties of the material, with 2 percent showing the best results. Taking into consideration previous researches, in the current study, we prepared GO/GIC hybrids containing 1 and 2% GO. Following that, we characterized their flexural strengths and compared them. Results indicate that 2% GO would significantly enhance the flexural strength of RMGI, while 1% GO did not have any significant effects. There was, however, no significant effect of the GO on CGIC. So, the null hypothesis was not confirmed.

GICs are often prone to failure due to voids and cracks in the matrix. Therefore, it is necessary to reinforce it with fillers to improve its mechanical performance and extend its longevity. Previous research has demonstrated that graphene and its derivatives can enhance the physical and mechanical properties of dental products. A combination of polymethyl methacrylate (PMMA) and GO nanoparticles enhanced the flexural strength and other mechanical properties of PMMA [18]. When incorporated into a matrix, GO has been shown to play a crucial role in crack deflection and bridging. This could potentially enhance the mechanical properties of PMMA [21]. It seems that the ability of GO to perform crack bridging, pulling-out, crack deflection, and crack tip shielding, can improve the mechanical features of RMGI as well as its flexural strength.

In a study, GO was compounded with a mineral clay called montmorillonite (MMt), consisting of aluminosilicate [19]. Fluoroaluminosilicate is one of the basic compounds in GIC. The adsorption performance of MMt is positively influenced by its hydrophilicity, swelling properties, and cation exchange capacity. The mineral clay minerals may also form electrostatic interactions and hydrogen bonds with the GO layers, allowing the preparation of hybrid materials or suspensions of GO and aluminosilicate that are well dispersed. In hybrid GO-MMt materials, typically formed by intercalation and costacking processes, the MMt phyllosilicate matrix stabilizes the carbonaceous layer structure as well as improves physicochemical properties. As mentioned, GIC has fluoroaluminosilicate as a basic compound, along with calcium, strontium, and lanthanum cations. Therefore, GIC provides a similar function to MMt and can form an improved material by combining it with GO. These explanations seem to justify the hybrid RMGI/GO's superiority.

The material GO is a biocompatible derivative of graphene that has oxygen-containing groups and can be used to improve the mechanical properties of scaffolds or nanocomposites. Unlike graphene, GO tends to be hydrophilic because of its functional groups (carboxyl, hydroxyl, and epoxy groups). One of the substances in GIC liquid is carboxylic acid, which exists in GO functional groups. It may cause the formation of a polymer matrix between the substances of RMGI and improve its flexural strength. GO also reinforces RMGI due to its presence of functional groups, which have the additional benefit of dispersing well in polar solvents. RMGI-GO improves the molecular-level dispersion and enhances the interfacial interaction, increasing flexural strength [22].

The RMGI flexural strength increase could be due to the following factors: In the first place, GO exhibits high intrinsic strength. Also, contributing to this improvement is the large specific surface area of the nanofiller and its rough surface, which can enhance matrix adhesion and interlocking [22]. Furthermore, the increased degree of hydration may have refined the pore structure [23]. The nanoscale crack propagation in these matrices is generally recognized to be suppressed by GO [24]. The crack branching mechanism that has been investigated in the previous study could also explain the flexural strength enhancement effect of graphene and graphene family materials. Crack bridging, pull-out, crack deflection, and crack tip shielding are the four main aspects of the mechanism [25]. In crack bridging, GO bridges the opposing surface and lessens the crack propagation force. A pull-out mechanism occurs when the shear force is greater than the interface strength, causing the graphene and matrix to stick together. Due to graphene's two-dimensional nanosheet structure, crack propagation occurs along a tortuous path, which dissipates a lot more energy due to the crack deflection. Due to insufficient energy, a crack that propagated to graphene became confined to the graphene sheet vicinity when it propagated to graphene sheets [20]. Due to this, it seems in our study that cracks in the RMGI matrix are expected to be arrested and bridged by GO and enhance its improvement.

It is important to take into consideration the fact that graphene is agglomerated in the matrix, which is one of the main disadvantages of using it as a reinforcement. It is hard for graphene sheets to homogeneously disperse into a matrix, which limits their application as mechanical reinforcement. As graphene hydrophobicity and van der Waals attraction are strong, this causes graphene sheets to aggregate into monolayered sheets that weakly interact, which affects the enhancement in the matrix [26, 27]. In our study, we combined GO and GIC mechanically, without using an intermediate chemical compound to stabilize their linkage. This would reduce the chance of creating a homogenous mixture of GIC/GO. It was obvious that agglomerated GO particles occurred during the mixing of powdered CGIC-GO with liquid. This may compromise the mechanical properties of the CGIC/GO hybrid, since the CGIC/GO samples did not demonstrate significant improvements in flexural strength in this study. We guess this hybrid probably needs more working time to increase its homogeneity and flexural strength. It is noteworthy that the RMGI/GO mixture did not show agglomerated particles of GO during mixing with liquid. Also, graphene has a very low bulk density, and handling the dry powder is difficult, which could explain why the flexural strength of GIC/GO was not improved in this study [28].

There was a study in which the mechanical properties of direct core build-up materials, including flexural strength, were compared, with silver cermet and RMGI [29]. It is noteworthy that silver cermet was introduced in order to enhance the properties of GIC. However, this study indicated that there was a lower value for all mechanical properties of cermet glass ionomers except for elastic modulus. The mean flexural strength of RMGI was 58.73 MPa, and the mean flexural strength of silver cermet was 45.79 MPa. Cermet has a dark color similar to the GIC/GO hybrid we examined in the current study, while RMGI containing 2% GO exhibits enhanced mechanical properties.

The flexural strength of RMGI is much higher than that of CGIC, according to previous studies [30, 31]. In comparison with the CGIC, the RMGI has a different nature. These RMGIs contain some methacrylate components that are common in resin composites, as well as a liquid portion containing a water-miscible monomer of methacrylate. Additionally, RMGIs contain free radical initiators that initiate the curing process in an additional chain reaction compared to CGICs, which only rely on the acid-base reaction between polycarboxylic acid and fluoroaluminosilicate glass, so RMGIs tend to have higher flexural strength than CGICs. It was found that the results of the current study were in line with the findings of previous studies.

In the present study, the liquid was applied by following the manufacturer's instructions. Due to improper fluid amounts in the mixture, the mechanical properties of the mixture may be altered; thus, GO's insignificant effect on CGIC could likely be caused by this. In addition, sufficient mixing time can affect the mechanical properties of the mixture [29, 32]. Our study followed the manufacturer's instructions concerning mixing time. A change in mixing time is likely to occur when GO is added to GICs. It may also be a factor causing the negative effect of GO when combined with GICs on flexural strength.

Besides, the water-powder ratio, humidity, and temperature are also factors that affect the physical properties [33, 34]. The current study standardized these conditions by preparing all the samples in one day. We followed the manufacturer's instructions for mixing the powder with liquid and preparing the samples at room temperature. Every sample was coated with a varnish layer to prevent early contamination of the GIC water and initial water absorption. On the surface of the samples, a transparent matrix strip was used to standardize all groups. It is also important to consider the storage environment when determining the flexural strength of GICs. It has been found that resin-based materials can be stored in distilled water and artificial saliva without affecting their micromorphology [35]. The samples were stored in distilled water at 37°C during the present study.

The limitation of this study was the lack of specimen exposure to the cyclic loading or thermal cycles, and conservation of the samples in saliva simulating the clinical conditions. Further studies with oral environment simulation and also evaluation of other physical properties after the incorporation of GO into GICs are suggested.

## 4. Conclusions

Regarding the limitations of the present study, it was concluded that

- The addition of 2% GO to RMGI, increases the flexural strength of RMGI.
- The flexural strength of RMGI is higher than that of CGIC.
- There is no significant difference between RMGI containing 1% GO and control RMGI.
- The addition of GO to CGIC has no significant effect on its flexural strength.

## Figures and Tables

**Table 1:** Materials used in the present study.

Materials	Country	Company	Composition
Conventional glass ionomer, fuji II	Japan	GC Corporation	Powder: fluoroaluminosilicate glass Liquid: polyacrylic acid, itaconic acid, tartaric acid, maleic acid, and water
Resin-modified glass ionomer, fuji II L C	Japan	GC Corporation	Powder: fluoroaluminosilicate glass Liquid: polyacrylic acid, 2-hydroxyl ethyl methacrylate, urethane dimethacrylate, camphorquinone, and distilled water
Nano-graphene oxide	India	KNVs Incorporation	Graphene oxide

**Table 2:** Mean flexural strength values and corresponding standard deviation.

Group	Mean flexural strength (MPa) ± SD
CGIC (control)	9.55 ± 4.22
CGIC + 1% GO	8.12 ± 4.61
CGIC + 2% GO	7.43 ± 4.08
RMGI (control)	21.21 ± 2.79
RMGI + 1% GO	23.43 ± 3.47
RMGI + 2% GO	26.82 ± 4.12

CGIC = conventional glass ionomer cement, RMGI = resin-modified glass ionomer, GO = graphene oxide.

**Table 3:** *P* values between groups (Tukey test).

Groups	CGIC (control)	CGIC + 1% GO	CGIC + 2% GO	RMGI (control)	RMGI + 1% GO	RMGI + 2% GO
CGIC (control)	—	0.963	0.832	0.001^*∗*^	0.001^*∗*^	0.001^*∗*^
CGIC + 1% GO	0.963	—	0.999	0.001^*∗*^	0.001^*∗*^	0.001^*∗*^
CGIC + 2% GO	0.832	0.999	—	0.001^*∗*^	0.001^*∗*^	0.001^*∗*^
RMGI (control)	0.001^*∗*^	0.001^*∗*^	0.001^*∗*^	—	0.802	0.027^*∗*^
RMGI + 1% GO	0.001^*∗*^	0.001^*∗*^	0.001^*∗*^	0.802	—	0.395
RMGI + 2% GO	0.001^*∗*^	0.001^*∗*^	0.001^*∗*^	0.027^*∗*^	0.395	—

CGIC = conventional glass ionomer cement, RMGI = resin-modified glass ionomer, GO = graphene oxide.

## Data Availability

The data that support the findings of this study are available from the corresponding author upon request.
